# Retrospective Observational Study of Nintedanib in Managing Idiopathic and Progressive Pulmonary Fibrosis in Routine Practice

**DOI:** 10.3390/jcm14186665

**Published:** 2025-09-22

**Authors:** Alessia Giovanna Andrisano, Nadia Castaldo, Francesco Giuliana, Davide Femia, Giuseppe Morana, Vincenzo Patruno, Giorgio Monteleone, Nicolò Reccardini, Rossella Cifaldi, Michael Hughes, Yukai Wang, Paola Confalonieri, Francesco Salton, Pietro Geri, Marco Confalonieri, Barbara Ruaro

**Affiliations:** 1 Pulmonology Unit, Cardiothoracic Department, Santa Maria Della Misericordia Hospital, 33100 Udine, Italy; 2 Department of Cardiovascular and Pulmonary Sciences, Catholic University of Sacred Heart, 00168 Rome, Italy; 3 Pulmonology Unit, Department of Medical Surgical and Health Sciences, Hospital of Cattinara, University of Trieste, 34149 Trieste, Italy; 4 Department of Rheumatology, Sheffield Teaching Hospitals NHS Foundation Trust, Royal Hallamshire Hospital, Sheffield S10 2JF, UK; 5 Department of Rheumatology and Immunology, Shantou Central Hospital, Shantou 515041, China

**Keywords:** pulmonary fibrosis (PF), idiopathic pulmonary fibrosis (IPF), progressive pulmonary fibrosis (PPF), interstitial lung diseases (ILDs), antifibrotic therapy (AT), nintedanib

## Abstract

**Background/Objectives:** Idiopathic pulmonary fibrosis (IPF) is the most common form of pulmonary fibrosis (PF) and serves as a key reference for disease severity. Progressive pulmonary fibrosis (PPF), a distinct yet heterogeneous entity arising from various interstitial lung diseases (ILDs), shares similar pathogenetic mechanisms and clinical courses driven by self-perpetuating fibrosis. Antifibrotic therapy, notably nintedanib, can slow disease progression. However, real-world data on antifibrotic therapy’s impact on survival, especially in PPF, are limited. This study aims to compare IPF and PPF regarding phenotype, radiological patterns, comorbidities, prognostic factors, and response to nintedanib, focusing on identifying the patient subsets most likely to benefit. Outcomes assessed include safety, survival, and disease progression over one year, considering various prognostic factors. **Methods:** This retrospective observational study evaluated patients with fibrosing ILD, affected by either IPF or PPF, and treated with nintedanib. Data collected encompassed clinical, radiological, functional, and treatment-related information. Assessments included chest CT, pulmonary function tests, comorbidities, and survival analysis, utilizing standardized methods and statistical tools to interpret outcomes and tolerability. **Results:** The study population was composed of 97 patients: 64 were diagnosed with IPF and 33 with PPF. The analysis showed that in PPF patients, ongoing antifibrotic treatment resulted in higher survival (71.1 months vs. 27.4 months, *p* < 0.001), while no statistically significant differences were found in the IPF group (67.4 months vs. 52.5 months, *p* = 0.216). Nintedanib was generally well tolerated. Gastrointestinal side effects, predominantly diarrhea, were reported in 61% of patients with IPF and 50% of those with PPF. Dose reduction occurred in 43.75% of IPF patients and 36% of PPF patients, while treatment discontinuation was required in 21.87% of IPF and 21% of PPF patients. **Conclusions:** This study highlights that in PPF patients, antifibrotic therapy with nintedanib can improve survival. This statement underlines that the primary outcome of antifibrotic treatment should focus on improving patients’ survival.

## 1. Introduction

Pulmonary fibrosis (PF) is the preferred general term for a wide group of diseases, known as interstitial lung diseases (ILDs), which primarily affect the pulmonary interstitium and can lead to the fibrotic remodeling of the lung tissue [1]. In genetically predisposed individuals (i.e., genetic polymorphisms and rare mutations), its pathogenesis encompasses a broad array of mechanisms such as focal or diffuse lung injury (mechanical, infectious, inflammatory, and iatrogenic) and, subsequently, abnormal lung repair that result in volume loss, architectural distortion, and lung failure [1,2]. Within the PF group, the natural history of these diseases can be heterogeneous over time, ranging from mainly inflammatory to predominantly fibrotic forms. Notably, while fibrotic ILDs (f-ILDs) are primarily fibro-proliferative disorders characterized by alveolar loss and fibroblastic proliferation that lead to lung fibrosis, other ILDs, predominantly inflammatory, are driven by underlying internal or external triggers that may start as inflammatory shifting to a fibro-proliferative pathway [1,3]. The f-ILD cluster includes a wide spectrum of diseases such as idiopathic pulmonary fibrosis (IPF), chronic fibrotic hypersensitivity pneumonitis (fHP), connective tissue disease-related ILDs (CTD-ILDs), sarcoidosis, genetic and familiar pulmonary fibrosis, unclassifiable ILDs (u-ILDs), idiopathic non-specific interstitial pneumonia (iNSIP), drug-induced ILD, interstitial pneumonia with autoimmune features (IPAF) and other ILDs [4]. Among them, IPF remains the one with the poorest prognosis at 3–5 years without antifibrotic treatment [5,6,7]. To date, nintedanib, an oral tyrosine kinase inhibitor, is an antifibrotic drug that has been approved for the treatment of IPF and progressive pulmonary fibrosis (PPF) [8,9]. This therapy was able to slow down the rate of lung function decline in patients with IPF as well as to prevent further progression and to reduce respiratory symptoms in patients with PPF once initial treatment had already failed [9,10,11,12,13,14]. Furthermore, it is well established that each ILD subtype carries a different risk of progression, and that risk stratification relies on the clinical, biochemical, molecular, physiological, histological, and/or radiological variables [15,16,17,18,19,20,21,22,23,24]. However, real-life data on the effectiveness of nintedanib on survival and clinical outcomes in settings of both IPF and PPF remain limited. Only a few studies have been conducted to assess the effectiveness of this antifibrotic treatment in both disorders [25,26,27,28,29,30,31,32,33,34]. Hence, we conducted a retrospective observational study to detect similarities and differences between IPF and PPF in terms of disease behavior, survival, and response to nintedanib, with the goal of defining the patient population most likely to benefit from antifibrotic therapy.

## 2. Materials and Methods

In this retrospective study, 97 consecutive patients diagnosed with IPF and PPF, and treated with nintedanib, were enrolled. Patients were enrolled at the Pneumology Department of the University Hospital of Trieste and the Pneumology and Respiratory Pathophysiology Department of the Hospital of Udine. The retrospective data collection was conducted during the months of October, November, and December, subsequent to obtaining approval from the ethics committee and in strict adherence to the timelines outlined in the retrospective study approval protocol. The diagnosis of IPF and PPF was performed in accordance with the current guidelines, based on clinical evaluation, lung function tests, chest high-resolution computed tomography (HRCT) scans, and multidisciplinary discussion (MDD) encompassing pulmonologists, radiologists, pathologists, rheumatologists, and cardiologists with experience in the field of ILDs [9]. Additionally, surgical lung biopsy was performed only in cases where the diagnosis remained uncertain following MDD. We aimed at investigating the safety and tolerance of both IPF and PPF patients to nintedanib, with particular attention to treatment-related side effects. The survival analysis was conducted to assess differences in mortality between IPF and PPF groups following nintedanib discontinuation in patients who underwent antifibrotic therapy for 6 months or longer. Patients who discontinued nintedanib or died within the first six months were excluded to allow for a more homogeneous analysis of long-term treatment outcomes. Moreover, annual rate of decline in forced vital capacity (FVC) and diffusing lung capacity for carbon monoxide (DLCO) were measured over a 52-week period in both IPF and PPF patients. Finally, the impact of prognostic factors (including age, sex, lung function decline over 52 weeks, radiological pattern, ILD gender/age/physiology score, nintedanib suspension) on survival was investigated.

### 2.1. Radiological Assessment

All patients recruited into this study underwent chest HRCT at baseline and at follow-up with a frequency variable between 6 months and 12 months from diagnosis. The chest HRCTs were, where possible, all performed in the same reference center, to avoid confounding factors linked to the different machinery in use and to allow evaluation by radiologists with expertise in the study of interstitial diseases. Each chest HRCT was reviewed in the context of MDD and, whenever diagnosis was made solely based on imaging, the pattern that received the greatest consensus from MDD was considered as definitive. Independently from which group they belonged to, patients were divided according to the radiological pattern into two subgroups: usual interstitial pneumonia (UIP) pattern and non-UIP pattern.

### 2.2. Respiratory Functional Assessment

Pulmonary function tests (PFTs), including FVC and DLCO assessments, were recorded at baseline (when treatment with nintedanib was established) and throughout the follow-up 6 and 12 months after therapy. When available, we also reported PFTs performed 12 and 6 months before therapy. Due to the retrospective nature of this study, functional tests conducted at these appropriate intervals were not always available. Therefore, we also considered as valid PFTs performed ±2 months from the established time. Lung function assessments were predominantly carried out using the same spirometer at the same recruitment center, to minimize variability related to equipment and operators’ differences.

To evaluate the impact of nintedanib on lung function, we calculated for every patient the slope between FVC and DLCO at baseline and FVC and DLCO at 12 months after treatment, and we compared the average decline in the two indices. We excluded patients who discontinued therapy before 12 months, and we included patients that at that time were still alive.

### 2.3. Assessment of Comorbidities

We screened all patients for comorbidities known to be associated with ILDs, including gastroesophageal reflux disease (GERD), chronic obstructive pulmonary disease (COPD), history of exposure to pneumotoxic agents, and lung cancer. Moreover, to evaluate the impact of comorbidities on survival, we calculated for every patient the CCI score; it encompasses history of myocardial infarction, chronic heart failure, peripheral vascular disease, cerebrovascular accident or transient ischemic attack, peptic ulcer disease, liver disease, diabetes mellitus, moderate to severe chronic kidney disease, and either solid or hematologic tumors.

### 2.4. Stage Assessment

The stage of pulmonary fibrosis was assessed through the ILD-GAP score and ILD-GAPC score, in order to evaluate the role of comorbidities on patients’ survival and to assess the precision of these scores in predicting mortality.

### 2.5. Statistical Analysis

Data were recorded in an electronic database. Statistical analysis was performed by using IBM SPSS Statistics 30.0. Continuous variables were assessed for normality using the Kolmogorov–Smirnov test and were expressed as the mean ± standard deviation. Categorical variables were expressed as frequencies and percentages. The parametric t-test was used to compare continuous variables and identify differences between the two groups. Survival analysis was conducted using Kaplan–Meier curves, and differences between groups were evaluated through the log-rank test. The rate of decline in FVC and DLCO was estimated by interpolating serial pulmonary function tests collected during the follow-up. FVC and DLCO declines were expressed as % of predicted values rather than milliliters to avoid the reduced sensitivity associated with the linear interpolation in cases where absolute decline in milliliters was often unavailable. The relationship between variables was assessed through multiple linear regression, and variability within the regression model was investigated by analysis of variance (ANOVA). A *p* value ≤ 0.05 was considered significant.

## 3. Results

A total of 97 patients affected with fibrosing ILDs (64 diagnosed with IPF and 33 diagnosed with PPF) and treated with nintedanib were retrospectively recruited; of them, 62 were followed by the Pneumology Department of the University Hospital of Trieste and 35 by the Pneumology and Respiratory Pathophysiology Department of the Hospital of Udine. Demographics, comorbidities, PFT, and chest HRCT pattern were investigated at baseline (Table 1).

### 3.1. Population

Within the IPF patients, 75% (*n* = 48) were males and the mean age at diagnosis was 72.27 ± 10.5. By contrast, PPF was diagnosed at a mean age of 67.79 ± 10.4, earlier than IPF (*p* = 0.025). In this group of patients, males and females were almost equally distributed and a prevalence of women (66.7%) was found only among patients with CTD-ILDs.

Nine patients were primarily affected by CTD-ILDs, six had iNSIP, five had fHP, five had IPAF, two had post-COVID-19 ILDs, two iatrogenic ILDs, and the remaining four were affected by other forms of pulmonary fibrosis (one fibrotic OP, one fibrotic sarcoidosis, one PAP, and one UC-ILD). The most represented category was that of CTD-ILDs (29%), among which there were four SSc-ILDs, three RA-ILDs, one Sjögren-related ILD, and one Ant synthetase syndrome.

### 3.2. Radiological Pattern

All patients underwent chest HRCT scans and were discussed in the MDD to define the radiological pattern. No semiquantitative analysis of lung involvement was performed. Among them, 89% (*n* = 57) had a definite or probable UIP pattern, while indeterminate for UIP and an inconsistent UIP pattern were found in 7 (10.9%) patients. In the PPF group, the UIP pattern, both definite and probable, was the prevalent (45%) followed by NSIP pattern (36.3%) and fHP (9%). Only three patients showed the presence of OP, u-ILD, and pulmonary alveolar proteinosis.

#### 3.2.1. Histological Pattern

In line with major guidelines, none of the patients with a definite UIP pattern on chest HRCT underwent lung biopsy. Among patients with a probable UIP pattern, lung biopsy was performed only in two of them, confirming a histological UIP pattern. Three individuals with indeterminate for UIP pattern at HRCT underwent lung biopsy, which confirmed the presence of a histological pattern compatible with UIP. Only two patients with PPF did not undergo lung biopsy due to the unfavorable risk/benefit ratio. In one of them the histological finding of NSIP pattern confirmed the primary diagnosis, while the others were non-specific fibrotic forms.

#### 3.2.2. BAL Cellularity

Nine patients underwent bronchoalveolar lavage (BAL) to detect cell differential counts and to rule out possible infectious agents before treatment was established. BAL cellular counts showed the following: macrophagic alveolitis in five of them, a neutrophil predominance in two of them, and lymphocytosis in one case in which diagnosis of IPF was still confirmed by cryobiopsy; in the remaining case BAL was not decisive due to poor cellularity in the sample. Within the eight PPF patients that underwent BAL, the most recurrent pattern was lymphocytosis (defined by the presence of at least 20% lymphocytes), which was detected in 75% of patients, followed by macrophage alveolitis, which was found in three patients.

#### 3.2.3. Comorbidities

Among 64 patients, 50 (77%) were current or previous smokers. Exposure to known pneumotoxic agents, such as asbestos and wood dust, was identified in 11 patients (17%). Other common comorbidities included gastroesophageal reflux (GERD) in 17 patients (27%), chronic obstructive pulmonary disease (COPD) in 8 individuals (12.5%), and pulmonary hypertension and lung cancer were found in 7 (11%) and 6 (9.5%) patients, respectively. In the PPF group a prevalence of current or previous smokers (37.25%) and patients suffering from GERD (42.4%) or pulmonary hypertension (15.1%) was found. Less frequent were exposure to pneumotoxic agents (15.1%), COPD (12.1%), and lung cancer (6%).

#### 3.2.4. Stage Assessment

According to the ILD gender/age/physiology score (GAP score), 40.6% of patients were at stage I, 48.4% at stage II, and 10.9% at stage III, while its form adjusted for comorbidities, named the ILD gender/age/physiology/comorbidities score (GAPC score), showed a prevalence of patients at stages II (50%) and III (42.2%) and only 5/64 patients at stage I. Regarding PPF, 93% of patients were at stages I and II according to the ILD-GAP score. However, most of them (79%) were found to be at stages II and III if screening with ILD-GAPC score corrected for the Charlson Comorbidity Index Score (CCIS). According to the second scoring system, only 7 out of 33 patients were at stage I (Table 2 and Table 3).

### 3.3. Treatment with Nintedanib in the Two Populations

#### 3.3.1. Subgroup Analysis: IPF

**Figure 1:**
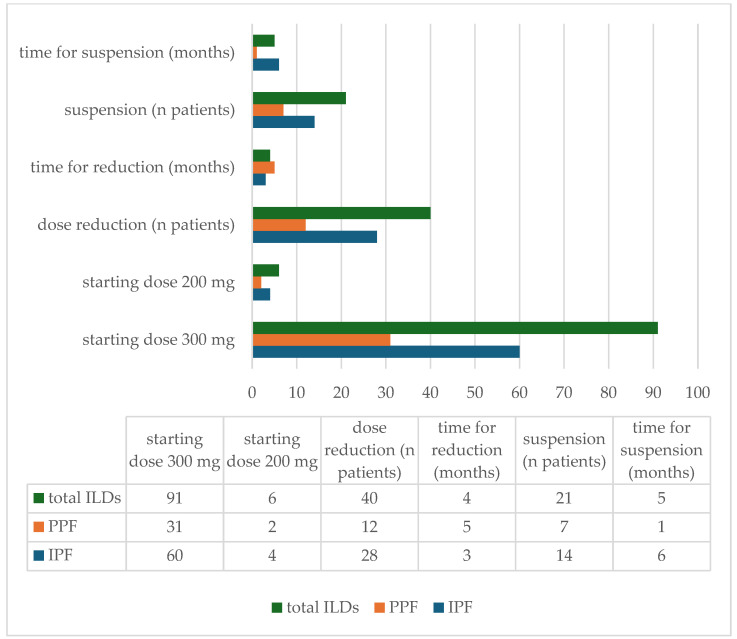
Variation in nintedanib dosage, time to dose reduction, and treatment discontinuation.

**Figure 2:**
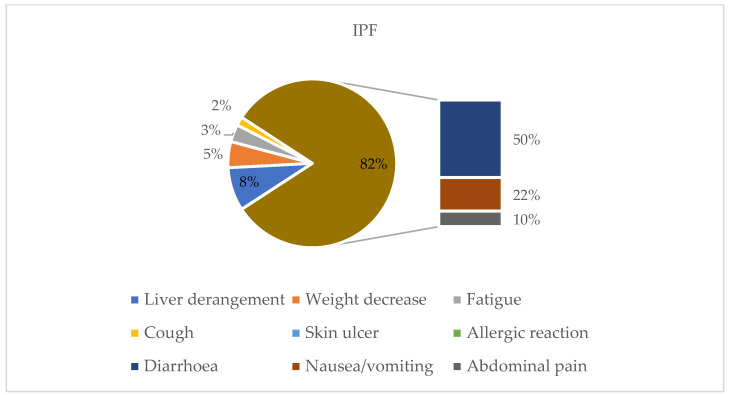
Side effects associated with nintedanib treatment in IPF patients. This figure shows the percentage of nintedanib side effects in the IPF group. Gastrointestinal diseases such as diarrhea (50%) and nausea and vomiting (22%) are the prevalent adverse effects, followed by liver derangement (24%) and abdominal pain (10%). Liver derangement and weight decrease were found in 8% and 5% of patients, respectively, while fatigue and cough were found in 3% and 2%.

**Figure 3:**
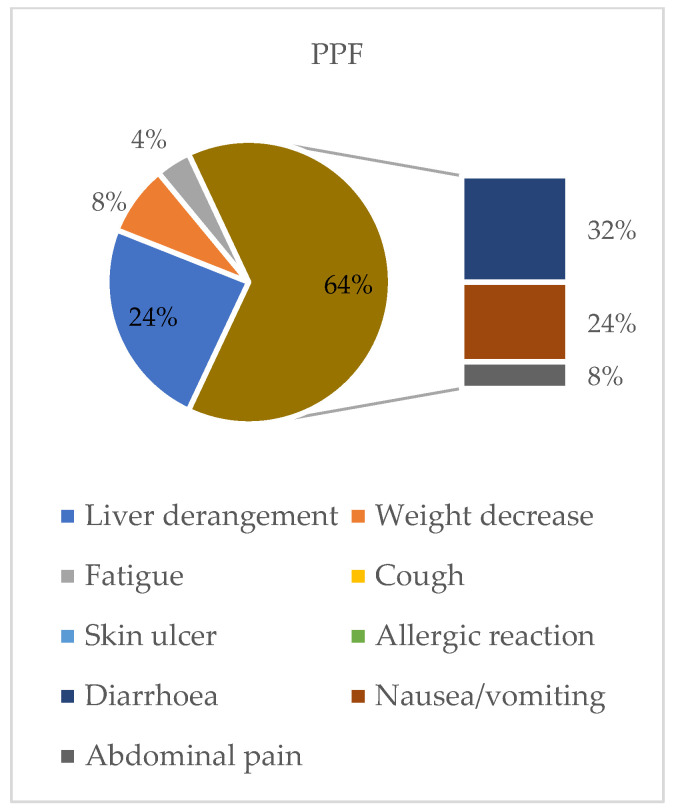
Side effects associated with nintedanib treatment in PPF patients. This figure shows the percentage nintedanib side effects in the PPF group. Gastrointestinal diseases such as diarrhea (32%) and nausea and vomiting (24%) are the prevalent adverse effects, followed by liver derangement (24%), abdominal pain and weight decrease (8%), and fatigue (4%).

Among the IPF group, treatment with nintedanib was established in 73% (*n* = 47), while it was established in 23% (*n* = 15) for ILD progression in patients previously treated with immunosuppressants that received an IPF diagnosis after MDD. Additionally, two of them started nintedanib following pirfenidone intolerance. Most patients (60/64) received the full dosage of 300 mg per day, while the remaining started with a decreased dose of 200 mg per day based on presumed intolerance, gastrointestinal or liver diseases, or known renal failure. Dose reduction was registered in 40 patients (28 IPF) with a median time of 4 months (5 months for PPF and 3 months for IPF). By contrast, 21 ILD patients (14 diagnosed with IPF) suspended the treatment and a median time of 5 months (6 months for IPF) for suspension was registered (Figure 1). The reason for dose reduction was gastrointestinal intolerance for most patients (82%) and the most common side effect was diarrhea (61%), followed by nausea/vomiting (27%) and abdominal pain (12%). A small percentage of patients experienced liver derangement (8%), weight loss (5%), fatigue (3%), and cough (2%), while no allergic reactions or skin ulcers were reported in any patient during treatment with nintedanib (Figure 2 and Figure 3). In total, 14 (21.87%) IPF patients discontinued the treatment; of them, 13 stopped antifibrotic therapy due to gastrointestinal intolerance, while only 1 stopped for lung transplantation (Figure 2 and Figure 3).

#### 3.3.2. Subgroup Analysis: PPF

Among the PPF group, nintedanib was introduced according to the guidelines when progression was demonstrated in at least two of the three (clinical, physiological, and radiological) domains within the past year, without alternative explanation [9]. In 36% of cases nintedanib was introduced in patients naive to immunosuppression, while in 64% of patients the antifibrotic drug was added when progression towards fibrosis was demonstrated despite other specific treatments for the primary diagnosis being established. Previous chronic oral steroid use was reported in 18 patients and the average daily dose was 10 mg per day. Steroids were administered alone or in combination with steroid-sparing agents within the CTD-ILD subgroup, in which all patients were treated with nintedanib and standard pharmacological management of CTDs, including immunosuppressive drugs, such as Methotrexate, Mycophenolate mofetil, Azathioprine, Hydroxychloroquine, and Rituximab, according to an MDD conducted with experienced rheumatologists.

Even in this case, almost all patients (31 of 33) received a full dosage of nintedanib of 300 mg per day, while only 2 patients received an initial daily dose of 200 mg. One third of patients with PPF (*n* = 12) had to reduce the initial dose after a median time of 5 months and 7 patients suspended the treatment after a median time of 1 month (Figure 1). Major reasons for dose reduction were gastrointestinal intolerance (64%) and liver derangement (24%). Moreover, 67% of patients did not tolerate the administration of both immunosuppressive therapy and nintedanib due to gastrointestinal adverse effects (Figure 3).

### 3.4. Survival Assessment

We recorded 33 deaths during the follow-up, 24 (72.7%) of which were from the IPF group and 9 (27%) of which were from the PPF group. The remaining 65% of overall patients were still alive at the end of observation.

In total, 14/33 dead patients had discontinued nintedanib before death occurred, and among all the patients who discontinued the drug, only 23.8% (5/21) were still alive at the end of observation.

Among the patients who discontinued treatment, death occurred in 57% of patients from the IPF group (8/14) and 85.7% of patients from the PPF group (6/7).

Survival rates were 89% at 12 months, 91% at 24 months, and 89% at 36 months among the overall population. In the IPF group, survival rates were 92.80%, 92.80%, and 86.7% at 12, 24, and 36 months, respectively, while in the PPF group survival rates were 95.90%, 97.90%, and 99%, respectively, during the same observation period.

**Figure 4:**
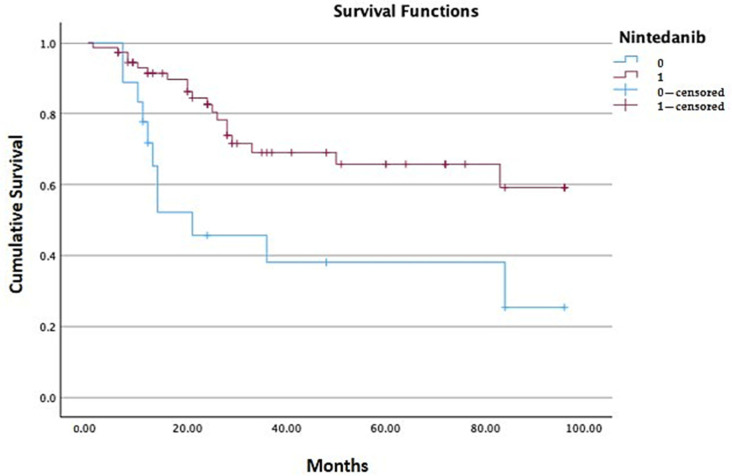
The estimated mean survival was 64.58 months among the overall population; among patients who discontinued nintedanib (level of nintedanib 0) the estimated mean survival is significantly reduced compared to patients with ongoing treatment (level of nintedanib 1) (44.47 versus 70.22 months, *p* = 0.004).

Among patients with ongoing nintedanib therapy, the percentage of the number of events was 25%, while in the group of patients who discontinued the drug death occurred in 58.8% of cases. In the overall population, the estimated mean survival of patients with ongoing nintedanib was 70.22 months, while patients who suspended the drug had a mean survival of 44.47 months, with a statistically relevant difference between the two subgroups (*p* = 0.004) [Table 4, Figure 4].

**Figure 5:**
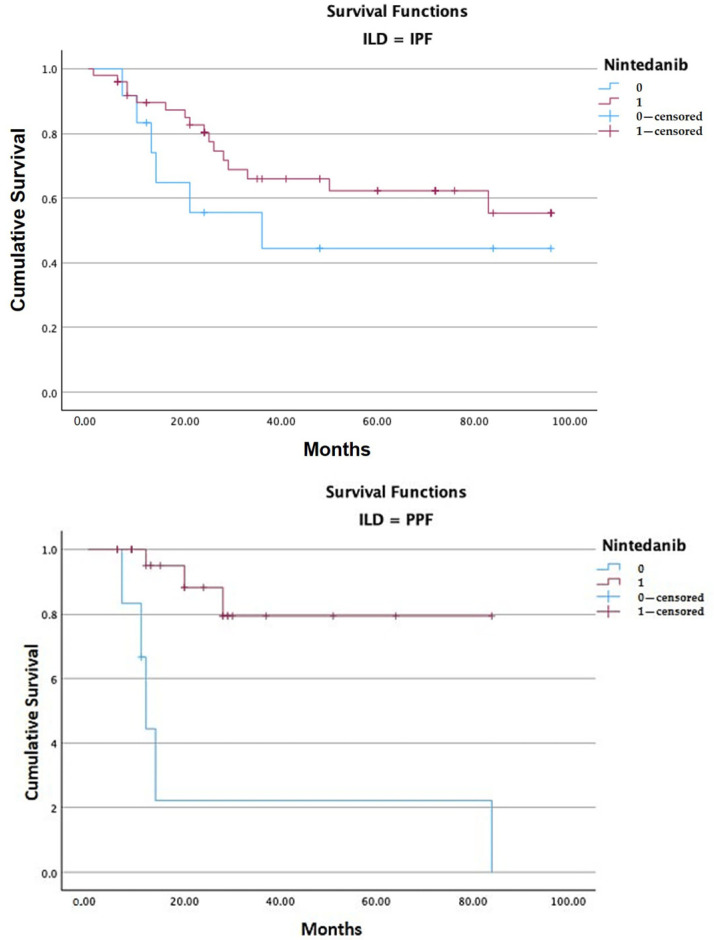
Kaplan–Meier analysis stratified for the IPF and PPF groups: in the IPF group the difference in estimated mean survival between patients who discontinued nintedanib (level of nintedanib 0) and patients who continued therapy (level of nintedanib 1) was not significant (*p* = 0.216), while in the PPF group, patients with ongoing nintedanib (level of nintedanib 1) had a significant higher estimated mean survival compared to patients who discontinued therapy (71.11 months versus 27.44 months, *p* < 0.001).

Stratifying patients into the two populations of study (IPF and PPF), only in the PPF group was there a statistically significant difference between patients with ongoing therapy and patients who discontinued nintedanib (*p* < 0.001) [Table 5, Figure 5]. In the IPF arm, even if estimated mean survival was higher in patients with ongoing nintedanib compared to patients who discontinued the drug, no statistical significance was found between the two groups (67.35 months versus 52.53 months, *p* = 0.216) [Table 5, Figure 5].

### 3.5. Impact of Nintedanib on Lung Function

Among the overall population treated with nintedanib, there was an increase in FVC % at 12 months of treatment of +0.51 ± 18.4%.

Among patients who discontinued therapy before 12 months, the mean annual decline in FVC was −4.41 ± 9.4%, which was faster but not significantly different from the mean annual decline in patients with ongoing therapy at 12 months (*p* = 0.115). In the IPF group, after 12 months of treatment FVC improved by +0.32 ± 20.5%, while in the PPF group it improved by +1.69 ± 13.3%, with no statistical significance between the two populations (*p* = 0.722) [Table 6].

The annual decline in DLCO among the overall population treated with nintedanib was −6.33 ± 15.7%, slightly more marked among the PPF group (−7.87 ± 15.5%) compared to the IPF group (−5.62 ± 15.8%), although no statistically significant difference was demonstrated between the two populations (*p* = 0.516). The annual decline in DLCO among patients who had already suspended the drug at 12 months was higher than the decline experienced by patients with ongoing nintedanib, although no statistically significant difference was demonstrated (−7.44 ± 15.7% vs. −6.10 ± 15.7%, *p* = 0.760) [Table 7].

### 3.6. Potential Predictors of Mortality in ILDs

In our study, we screened patients for the previously mentioned negative prognostic factors (advanced age at diagnosis, male sex, UIP pattern on HRCT, longitudinal decline in FVC % after 12 months of treatment with nintedanib) and for the most common comorbidities associated with ILDs, and we lastly derived ILD-GAP and ILD-GAPC scores for each patient [Table 3], in order to detect which prognostic factor or which of the two scoring systems better correlates with mortality. Moreover, we included nintedanib suspension as a potential risk factor to assess if it would cause greater risk of early mortality.

This study showed that ILD-GAPC score was more predictive than ILD-GAP score and that the presence of multiple negative parameters at the same time, including advanced age at diagnosis, male sex, UIP pattern on HRCT, and longitudinal decline in FVC (%) after 12 months of treatment, in addition to nintedanib suspension, could cumulatively influence early mortality (*p* = 0.002) in all ILDs.

In the IPF population, stage III PF for the ILD-GAPC score was the greatest predictor of mortality (*p* = 0.043), while nintedanib suspension did not significantly increase risk of mortality. By contrast, in the PPF group, nintedanib suspension and advanced age at diagnosis (*p* < 0.001) were the variables that most strongly predicted mortality [Table 8].

## 4. Discussion

Our study provides valuable real-world insights into survival, disease progression, and response to antifibrotic therapy with nintedanib in both IPF and PPF patients. When contextualized with the existing literature, several findings emerge that both align with and extend current knowledge.

Firstly, the average age at ILD diagnosis in our cohort was 70.74 ± 10.7 years, notably older than the mean age reported in prior studies, which typically ranges from 65 to 70 years [16,17]. This discrepancy could reflect regional demographic differences or evolving diagnostic practices. Interestingly, PPF patients were significantly younger at disease onset (mean age 67.79 ± 10.4) than IPF patients, aligning with previous reports indicating that PPF can present across a broader age spectrum, often younger than classic IPF [18].

Gender distribution in our cohort—male predominance in IPF and female prevalence in CTD-ILDs—corroborates established epidemiological patterns documented in prior studies [6,19]. Regarding radiological patterns, UIP was present in 46% of PPF patients and was the most common pattern on HRCT across the entire population. This finding supports prior research indicating that UIP is not exclusive to IPF but also frequently observed in other fibrotic ILDs, such as chronic hypersensitivity pneumonitis (cHP) and RA-ILD [20]. Hence, this highlights that the finding of a UIP pattern may require a careful assessment to optimize its management.

The high prevalence of comorbidities such as GERD, COPD, lung cancer, and pulmonary hypertension mirrors previous large cohort studies [8,21], emphasizing their significance in the clinical course of ILDs, especially in IPF. Notably, GERD’s role as a potential contributor to disease progression remains debated, but its frequent co-occurrence underscores the importance of comprehensive management strategies [22].

Regarding antifibrotic therapy, our findings align with current guidelines, which recommend nintedanib for IPF and, more recently, for PPF with progressive features. In our real-world setting, nintedanib was generally well tolerated and was discontinued by 20 patients (20.6%), similar to reported rates in the INPULSIS trials [8]. Gastrointestinal side effects, primarily diarrhea, were the most common and often manageable, consistent with previous observational studies [17,23,24,25,26,27,28,29,30,31,32,33,34,35,36,37,38,39,40,41,42,43,44,45,46,47,48,49,50,51,52,53,54].

Our analysis showed that the patients who maintained therapy exhibited a mortality rate of 25% versus 58.8% among those who discontinued it. This is in line with the findings from the CZECH EMPIRE registry where IPF patients treated with nintedanib had longer survival compared to those without treatment [24,55,56,57,58]. The mean survival of 70.22 months in patients continuing therapy compares favorably with previous real-world data, which report median survival extending beyond 3–4 years.

In the PPF subgroup, the survival difference between patients continuing versus discontinuing nintedanib was particularly pronounced (71.11 vs. 27.44 months, *p* < 0.001), highlighting the potential benefit of sustained therapy in this heterogeneous population. Although data from the INBUILD trial demonstrated that nintedanib slowed disease progression in PPF patients with a median follow-up of 17.6 months, the role of nintedanib in improving patients’ survival in PPF patients is still debated [25,26,27].

Our multivariate analysis identified nintedanib suspension and stage III ILD (per ILD-GAP score) as strong predictors of mortality across all ILD patients. This aligns with prior studies emphasizing the prognostic value of ILD-GAP indices [28,29], and underscores the importance of therapy adherence. Furthermore, traditional predictors such as age, sex, UIP pattern, and FVC decline did not consistently reach significance, echoing recent research suggesting that composite scores and treatment status may be more informative.

Lung function trajectories showed modest improvements or stabilization with nintedanib, consistent with the INPULSIS trials and world data from a Dutch registry on IPF and PPF where FVC decline was attenuated but not reversed [8,30]. In another study, Raman et al. observed that nintedanib slows down lung function impairment by attenuating FVC and DLCO declines in PPF patients [27]. The observed mean increase in FVC (+0.51%) and the non-significant difference compared to early discontinuers mirror findings by several studies, suggesting that while nintedanib may help stabilize lung function, individual responses can vary considerably, particularly in heterogeneous populations such as PPF. Although we observed that there were no significant differences in lung function parameters between the patients who continued nintedanib and those who stopped antifibrotic therapy, the treatment with nintedanib appeared to improve PPF patients’ survival. This finding may be due to the multitarget effects of nintedanib by interfering with fibrogenesis pathways as well as the difference in disease severity, number of comorbidities, and responsiveness to treatment that are not evident from the lung function assessment alone. Hence, it suggests the need for a reliable biomarker of disease progression in both IPF and PPF to achieve a better management of this wide array of diseases. Moreover, it reflects that both IPF and PPF are determined by multi-pathways that cooperate in determining fibrosis progression over a period of time that differs according to disease characteristics, comorbidities, and response to treatment. The limitations of our study include the relatively small sample size, especially in the PPF group, reflecting the recent adoption of this concept and therapy in clinical practice. The reliance on HRCT, moreover, limited by the lack of a quantitative radiological assessment, and on MDD without histological confirmation introduces potential misclassification, as previous studies have shown that histology can refine diagnosis and management. A major limitation is also represented by the potential for indication and reverse causation bias: patients with more advanced disease may discontinue treatment due to worsening clinical status, rather than treatment discontinuation leading to worse outcomes. Furthermore, the lack of longitudinal PFT data prior to therapy limits definitive conclusions about the true impact of nintedanib on disease progression. In addition, treatment duration among patients who discontinued is heterogeneous among our population, which could have a strong impact on the interpretation of survival assessment.

## 5. Conclusions

This real-world study provides meaningful insights into the clinical characteristics, treatment, and clinical outcomes of patients with IPF and PPF receiving antifibrotic treatment with nintedanib. Our findings support and extend the existing evidence, showing nintedanib is generally well tolerated and may contribute to improved survival in patients with both IPF and PPF. These results suggest that the benefits of nintedanib may extend beyond spirometry stabilization due to its broader antifibrotic effects on multiple disease pathways. The finding that nintedanib discontinuation and advanced ILD-GAP stage are key predictors of mortality highlights the importance of sustained treatment and timely disease staging. Although age, FVC decline, and UIP pattern are prognostic factors with a well-established role, particularly in IPF, there remains a clear need for more sensitive and specific biomarkers to guide clinical decision-making, especially within the heterogeneous field of PPF. However, given the observational nature of this study and the lack of significant differences in lung function trajectories, these results should be interpreted with caution, as reverse causation and indication bias may have influenced outcomes.

Despite certain limitations, including relatively small sample size, absence of histopathological confirmation, and variability in treatment duration, this study enhances the complexity of fibrotic ILDs and multifactorial mechanisms driving disease progression. Moving forward, larger prospective studies integrating molecular and imaging biomarkers are essential to improve patients’ stratification and guide personalized treatment approach across the ILD spectrum. Future prospective studies with larger cohorts and comprehensive histological data are needed to better delineate predictors of response and optimize individualized treatment strategies.

## Figures and Tables

**Table 1:** Demographics, comorbidities, pulmonary functions tests, and high-resolution computed tomography pattern at baseline of study population.

	*n* = 97	IPF = 64	PPF = 33
Gender (F/M) *n*, %	65/32	48 (75%)/16 (25%)	17 (51%)/16 (49%)
Age, years (mean ± SD)	70.74 ± 10.7	72.27 ± 10.5	67.79 ± 10.4
Smoking status (never/ex, current) *n*, %	28 (28.9%)/69 (71.1%)	50 (77%)	19 (37.25)
**Comorbidities**			
Gastroesophageal reflux (GERD) *n*, %	31 (31.9%)	17 (26.5%)	14 (42.4%)
Chronic obstructive pulmonary disease (COPD) *n*, %	12 (12.4%)	8 (12.5%)	4 (12.1%)
Pulmonary hypertension	12 (12.4%)	7 (11%)	5 (15.1%)
Lung cancer	8 (8.2%)	6 (9.3%)	2 (6%)
Exposure to pneumotoxic agents (drugs; asbestos; molds; wood dusts) *n*, %	16 (16.5%)	11 (17.1%)	5 (15.1%)
**HRCT pattern**			
UIP (definite, probable) *n*, %	72 (74.2%)	57 (89%)	15 (45.4%)
Indeterminate or inconsistent for UIP *n*, %	7 (7.2%)	7 (10.9%)	-
NSIP *n*, %	12 (12.3%)	-	12 (36.3%)
fHP *n*, %	3 (3%)	-	3 (9%)
OP *n*, %	1 (1%)	-	1 (3%)
PAP *n*, %	1 (1%)	-	1 (3%)
u-ILD *n*, %	1 (1%)	-	1 (3%)
**PFT at baseline** (mean ± SD)			1 (1.96)
FVC %pred	-	80.2 ± 20.8	71.4 ± 21.5
DLCO %pred	-	47.6 ± 18.5	53.4 ± 30.1

**Table 2:** ILD-GAP and ILD-GAPC model scores. ILD-GAP score is a revised version of the GAP model that is not limited to IPF since it was developed for application across all ILD subtypes; additionally, for the traditional parameters (gender, age, FVC %, and DLCO % predicted), it also includes ILD diagnosis, thus providing cause-specific survival estimates. ILD-GAPC score is a novel model that combines the ILD-GAP model with the Charlson Comorbidity Index Score (CCIS), a summed score of 19 comorbidities weighted according to severity, which could also be a prognostic indicator in patients with ILDs [35,36].

		ILD-GAP Model Score	ILD-GAPC Model Score
ILD diagnosis	IPF/UC-ILD ^1^	0	0
	CVD-IP ^2^ ± iNSIP ^3^ ± CHP ^4^	−2	−2
Gender	Female	0	0
	Male	1	1
Age	≤60	0	0
	61–65	1	1
	>65	2	2
% FVC ^5^	>75	0	0
	50–75	1	1
	<50	2	2
%DLCO ^6^	>55	0	0
	36–55	1	1
	≥35	2	2
	Cannot perform	3	3
CCIS ^7^	0–1		0
	2–3		1
	≥4		2

^1^ Unclassifiable Interstitial Lung Disease. ^2^ Collagen Vascular Disease-related Interstitial Pneumonia. ^3^ Idiopathic Non-Specific Interstitial Pneumonia. ^4^ Chronic Hypersensitivity Pneumonitis. ^5^ Percentage Predicted Forced Vital Capacity. ^6^ Percentage Predicted Diffusing Capacity of the Lungs for Carbon Monoxide. ^7^ Charlson Comorbidity Index Score.

**Table 3:** ILD-GAP and ILD-GAPC models applied to our patients.

	IPF	PPF
**ILD-GAP score**		
Stage I	26	16
Stage II	31	15
Stage III	7	2
**ILD-GAPC score**		
Stage I	5	7
Stage II	32	13
Stage III	27	13

**Table 4:** Survival analysis among the overall population.

Nintedanib	N Patients	N Deaths	Mean Estimated Survival ^2^
0 = suspended	18	11	44.477 (CI 25.56–63.39)
1 = ongoing	76	19	70.228 (CI 60.63–79.82)
Total population ^1^	94	30	64.588 (CI 55.72–73.45)
**Overall Comparisons**	Chi-Square	Df	Sig.
Log Rank (Mantel–Cox) ^3^	8.260	1	0.004

^1^ The number of ILDs considered is 94 instead of 97 because the number of months until death occurred was unknown for three patients who died. ^2^ Expressed in months. Estimation is limited to the largest survival time if it is censored. ^3^ Test of equality of survival distributions for the different levels of nintedanib (0 = ongoing versus 1 = suspended).

**Table 5:** Survival analysis stratified by the groups in this study (IPF and PPF).

	Nintedanib	N Patients	N Deaths	Mean Estimated Survival ^1^
IPF	0 = suspended	12	6	52.520 (CI 28.72–76.33)
	1 = ongoing	50	16	67.355 (CI 56.08–78.63)
	total IPF	62	22	64.267 (CI 53.92–74.62)
PPF	0 = suspended	6	5	27.444 (CI 0.00–58.02)
	1 = ongoing	26	3	71.117 (CI 57.86–84.37)
	total PPF	32	8	62.054 (CI 47.33–76.78)
**Overall Comparisons**		Chi-Square	Df	Sig.
IPF	Log Rank (Mantel–Cox) ^2^	1.532	1	0.216
PPF	Log Rank (Mantel–Cox)	12.174	1	<0.001

^1^ Expressed in months. ^2^ Test of equality of survival distributions for the different levels of nintedanib (0 = ongoing versus 1 = suspended).

**Table 6:** *t*-test for annual FVC % decline.

	Nintedanib After 1 Year of Observation	N Patients	Mean Difference (FVC % at Baseline—FVC % at 1 Year)	Sig.
All	0 = suspended	17	−4.41 ± 9.4%	t 1.606, df 46 *p* = 0.115
	1 = ongoing	80	0.51 ± 18.4%	
IPF		53	0.32 ± 20.5%,	t −0.357, df 71 *p* = 0.722
PPF		26	1.69 ± 13.3%	

**Table 7:** *t*-test for annual DLCO % decline.

	Nintedanib After 1 Year of Observation	N Patients	Mean Difference (DLCO % at Baseline—DLCO % at 1 Year)	Sig.
All	0 = suspended	78	−7.44 ± 15.7%	t 0.309, df 21 *p* = 0.760
	1 = ongoing	16	−6.10 ± 15.7%	
IPF		64	−5.62 ± 15.8%	t −0.654, df 57 *p* = 0.516
PPF		30	−7.87 ± 15.5%	

**Table 8:** Predictors of mortality in the overall population (ANOVA).

Predictors *	IPF	PPF	All ILDs
Advanced age at diagnosis		X ^2^	
Male gender			
UIP pattern HRCT			
FVC % decline 1 year after nintedanib introduction			
Stage III pulmonary fibrosis according to ILD-GAP score			
Stage III pulmonary fibrosis according to ILD-GAPC score	X ^1^		X ^4^
Nintedanib suspension		X ^3^	X ^5^

* Death is the dependent variable. ^1^ F = 4.25, *p* = 0.043. ^2^ F = 17, *p* < 0.001. ^3^ F = 27, *p* < 0.001. ^4^ F = 10.46, *p* < 0.001. ^5^ F = 14.35, *p* < 0.001.

## Data Availability

All the data are available upon reasonable request to the corresponding author.

## Abbreviations

The following abbreviations are used in this manuscript:

ILD	Interstitial Lung Disease
IPF	Interstitial Pulmonary Fibrosis
PPF	Progressive Pulmonary Fibrosis
PF	Pulmonary Fibrosis
UIP	Usual Interstitial Pneumonia
GERD	Gastroesophageal Reflux
COPD	Chronic Obstructive Pulmonary Disease
CTD-ILD	Connective Tissue Disease-related Interstitial Lung Disease
iNSIP	Idiopathic Non-Specific Interstitial Pneumonia
UC-ILD	Unclassifiable Interstitial Lung Disease
fHP	Fibrotic Hypersensitivity Pneumonitis
IPAF	Interstitial Pneumonia with Autoimmune Features
OP	Organizing Pneumonia
PAP	Pulmonary Alveolar Proteinosis
SSc-ILD	Systemic Sclerosis-related Interstitial Lung Disease
RA-ILD	Rheumatoid Arthritis-related Interstitial Lung Disease
